# Jejunal Perforation Caused by Accidental Denture Swallowing: A Case Report

**DOI:** 10.7759/cureus.101060

**Published:** 2026-01-08

**Authors:** Abdullah H Marei, Ahmed G Hassan, Marwan E Makayed, Ahmed Abouelseoud, Yasser M Hamza

**Affiliations:** 1 General Surgery, Faculty of Medicine, Alexandria University, Alexandria, EGY; 2 Faculty of Medicine, Alexandria University, Alexandria, EGY

**Keywords:** acute abdomen, denture ingestion, exploratory laparotomy, foreign body ingestion, intestinal perforation, jejunal perforation, small bowel obstruction

## Abstract

Accidental denture ingestion, though rare, is a serious cause of acute abdomen. While most swallowed dentures pass through without complications, some can become impacted, leading to potentially severe complications. Diagnosis is challenging due to the radiolucent nature of dentures. We present a rare case of denture impaction in the jejunum, resulting in intestinal obstruction and perforation.

A 70-year-old male presented with diffuse abdominal pain, vomiting, and constipation after accidentally swallowing part of his dentures 10 days earlier. The patient sought medical advice three and five days before the current presentation, during which multiple non-contrast CT scans were performed and demonstrated the presence of dentures within the small bowel without evidence of obstruction or perforation. However, a subsequent contrast-enhanced CT scan revealed jejunal dilatation, a hyperdense structure, and a small bowel feces sign, consistent with mechanical obstruction. The hyperdense structure was identified as the metallic component of the ingested denture. Emergency laparotomy revealed jejunal perforations, and jejunal resection with end-to-end anastomosis was performed. The patient recovered well and was discharged after seven days.

Foreign body ingestion can occur in both children and adults, with fish bones, bones, and dentures being commonly swallowed by adults. Dentures, made of radiolucent materials, can be challenging to detect on plain radiographs, necessitating high clinical suspicion and the use of CT scans for accurate diagnosis. While denture impaction is more common in the esophagus or large bowel, cases in the small bowel are extremely rare. Complications such as perforation can occur, especially within a week, if the foreign body does not pass spontaneously. Conservative management is initially recommended, but surgical intervention may be necessary, particularly for lower gastrointestinal tract foreign bodies.

Elderly patients with acute abdominal symptoms and denture ingestion history should be suspected of complications. A high index of suspicion and prompt contrast-enhanced CT are crucial. Surgical intervention should not be delayed for impacted lower GI tract foreign bodies to prevent life-threatening complications like perforation.

## Introduction

Acute abdominal pain is a frequently encountered complaint in emergency departments, and it can have a wide range of causes, from minor to severe. Foreign body ingestion is a rare but serious cause of acute abdominal pain. In most cases, swallowed foreign bodies pass through the digestive system without any problems and only require observation. However, in a few rare instances, serious complications can occur, including bleeding, tissue necrosis, bowel obstruction, perforation, and even penetration into nearby organs, leading to fistula formation [1,2].

Artificial teeth, including crowns, implants, and dentures, account for 4% to 18% of cases. Moreover, removable partial dentures are the single most commonly reported in elderly patients [3]. On the rare occasion that dentures become impacted, it most commonly occurs in the esophagus, followed by the large bowel [4]. However, perforation can happen at any site along the gastrointestinal tract, although angulated bowel segments, including the rectosigmoid or ileocecal junctions, are the most common sites [5].

Diagnosing denture ingestion is challenging due to their radiolucent nature, and unsuccessful attempts to create radiopaque dentures without compromising quality make timely detection difficult. As such, contrast-enhanced computerized tomography (CT) remains the gold standard for identifying ingested dentures and assessing complications [6].

While numerous cases of denture impaction in the aerodigestive tract have been described [4], small bowel impaction remains exceedingly rare. We present a unique case of jejunal denture impaction complicated by mechanical obstruction and subsequent perforation, highlighting the diagnostic challenges associated with radiolucent foreign bodies and the importance of maintaining a high index of suspicion in patients presenting with acute abdominal pain following denture ingestion.

## Case presentation

A 70-year-old male presented to the emergency department with a 10-day history of diffuse abdominal pain, progressive abdominal distension, persistent vomiting, and absolute constipation. The patient reported accidentally swallowing a part of his dentures and had previously visited the emergency department two separate times, three and five days before the current presentation, with similar complaints. Non-contrast CT scans conducted during those visits revealed the dentures as an intraluminal hyperdense foreign body within the small bowel loops, along with minimal fat stranding. However, there were no significant loculated collections, free pneumoperitoneum, or intraperitoneal fluid. The bowel loops appeared of normal size without signs of intestinal obstruction or perforation. Based on these findings, it was believed that the dentures would pass through the gastrointestinal tract and be evacuated during defecation, leading to the patient's discharge on both occasions.

The patient also disclosed ingesting around 500 g of cotton in an attempt to facilitate transit. This action was not medically advised and was undertaken independently by the patient. The patient had no notable past medical, psychiatric, or surgical history.

Upon examination, the patient exhibited visible distress and pain. Vital signs revealed a blood pressure of 130/70 mmHg, heart rate of 130 beats per minute, respiratory rate of 32 breaths per minute, and oxygen saturation of 99% on room air. The patient was afebrile, with an oral temperature of 37.2 °C. Abdominal examination showed generalized tenderness without rebound tenderness or rigidity, and bowel sounds were increased. Per rectal examination revealed an empty rectum.

Laboratory results demonstrated a white blood cell count of 6.2 × 10^9^/L, hemoglobin level of 12.5 g/dL, platelet count of 232 × 10^9^/L, sodium level of 142 mEq/L, and potassium level of 4.9 mEq/L. Contrast-enhanced CT scan of the abdomen and pelvis revealed dilation of most jejunal bowel loops, measuring 3.3 cm in caliber, with a transitional zone observed at the distal jejunum/proximal ileum containing an intraluminal hyperdense structure. The presence of the small bowel feces sign (SBFS) was noted. Distally, the ileal loops appeared relatively collapsed, and the large bowel loops were of normal size. These findings were indicative of mechanical small bowel obstruction.

**Figure 1:**
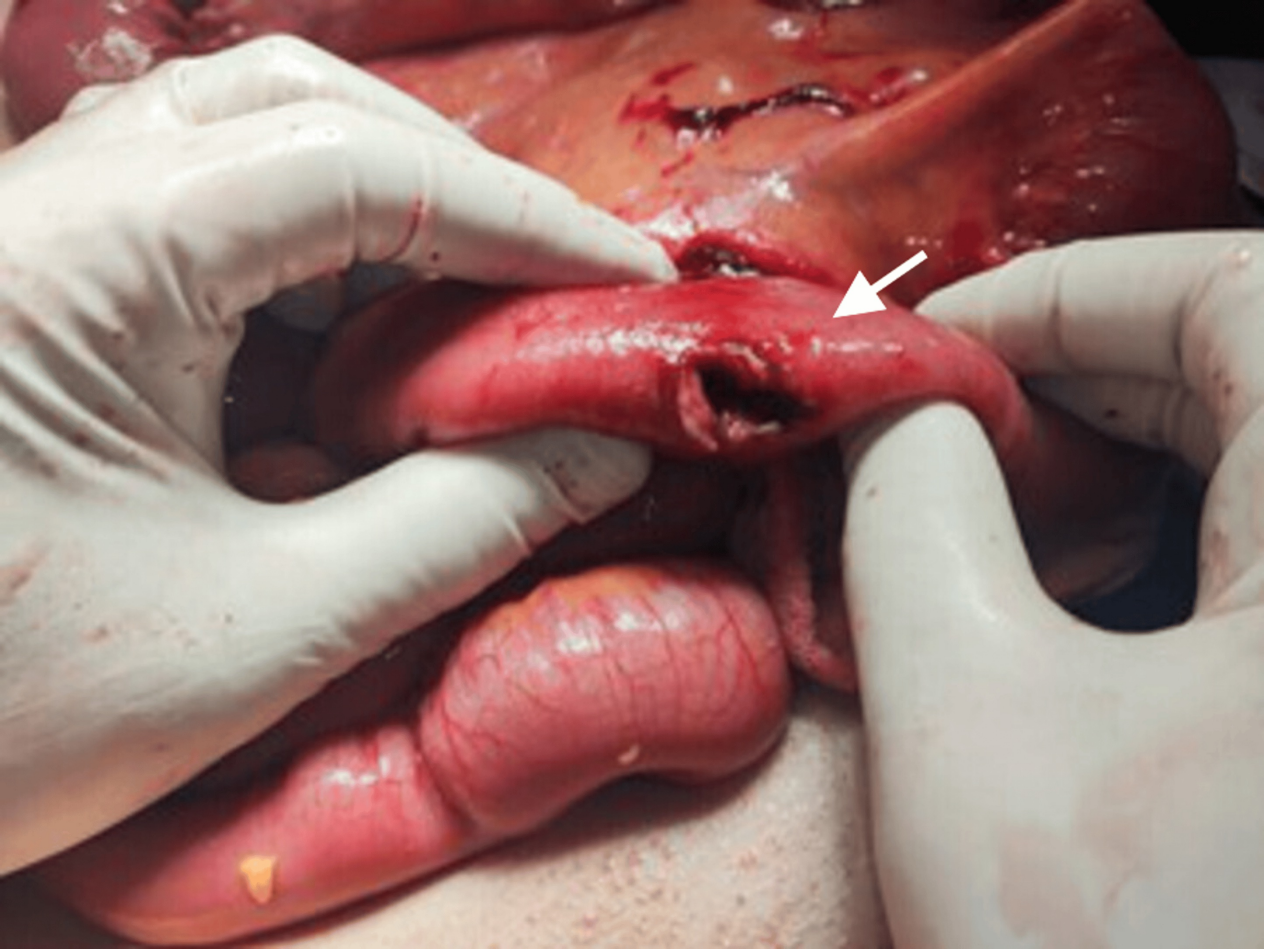
Intraoperative image demonstrating the first jejunal perforation located approximately 1 m distal to the duodenojejunal flexure.

**Figure 2:**
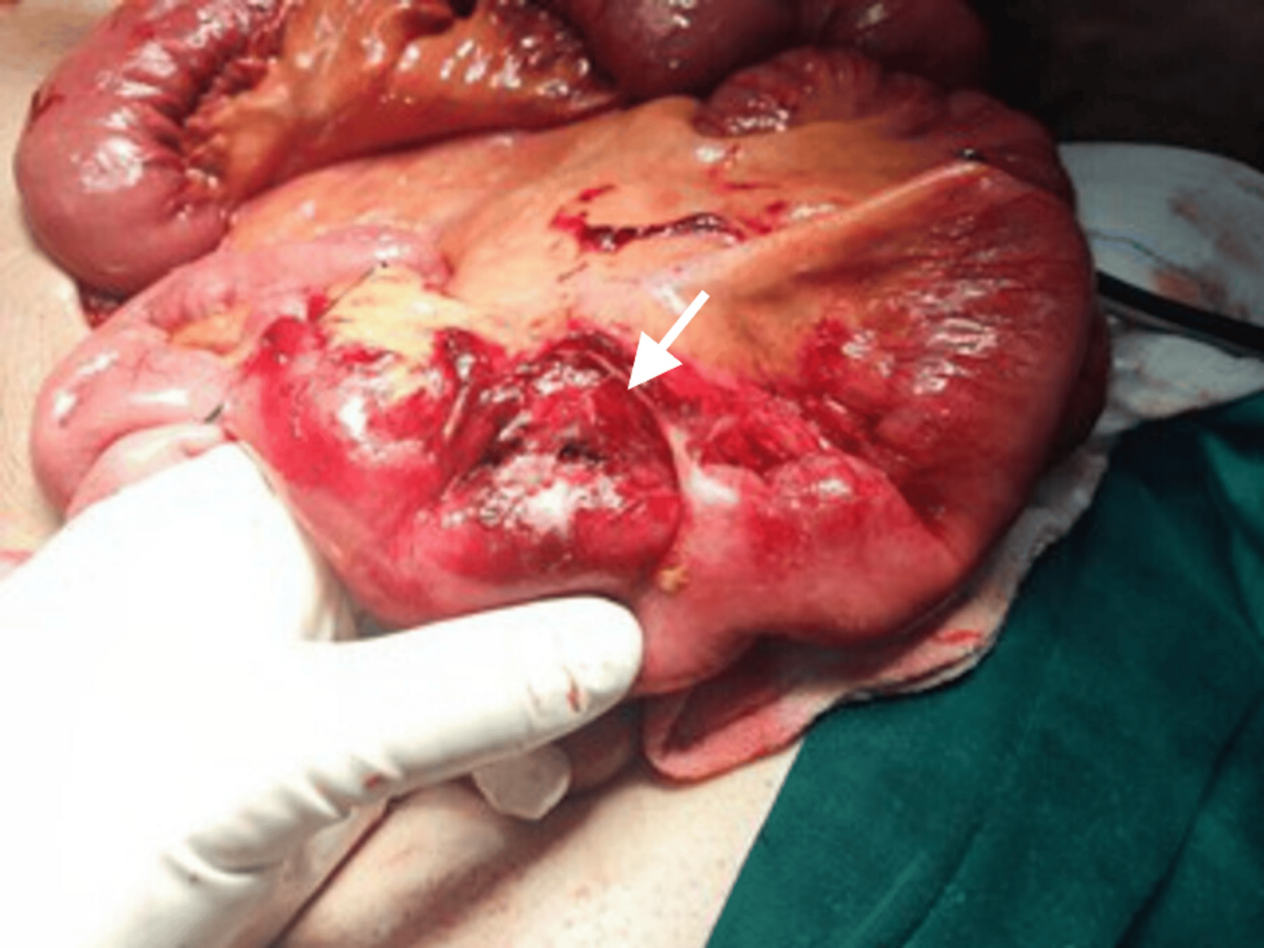
Intraoperative view showing a second jejunal perforation identified during surgical exploration.

The patient was promptly resuscitated, initiated on intravenous antibiotics, and scheduled for an emergency exploratory midline laparotomy. Intraoperative exploration revealed two jejunal perforations, located approximately one meter distal to the duodenojejunal flexure (Figures 1-2), with localized contamination.

**Figure 3:**
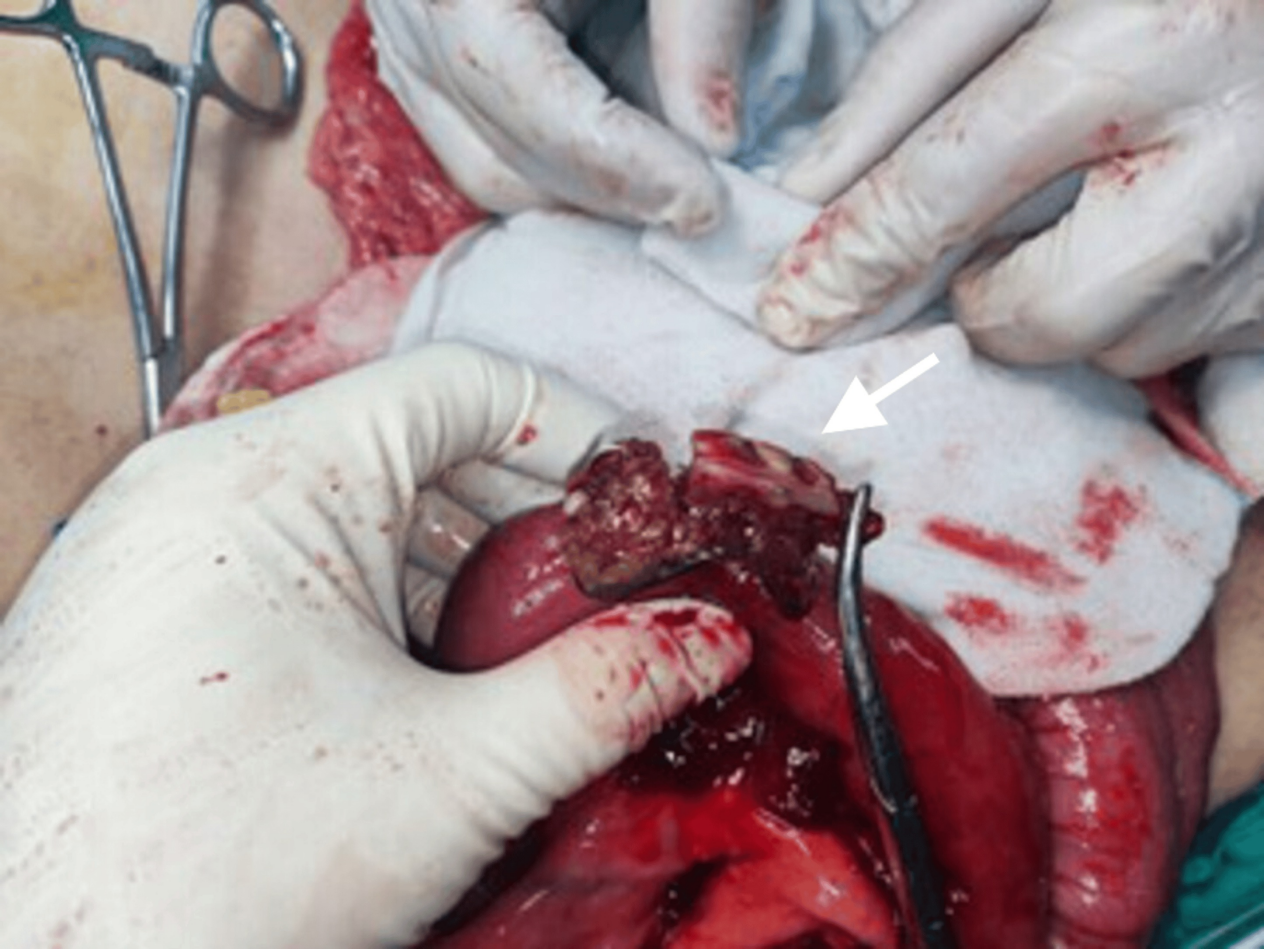
Intraoperative view of the ingested denture. An intact partial denture, along with the ingested cotton, identified within the jejunal lumen.

**Figure 4:**
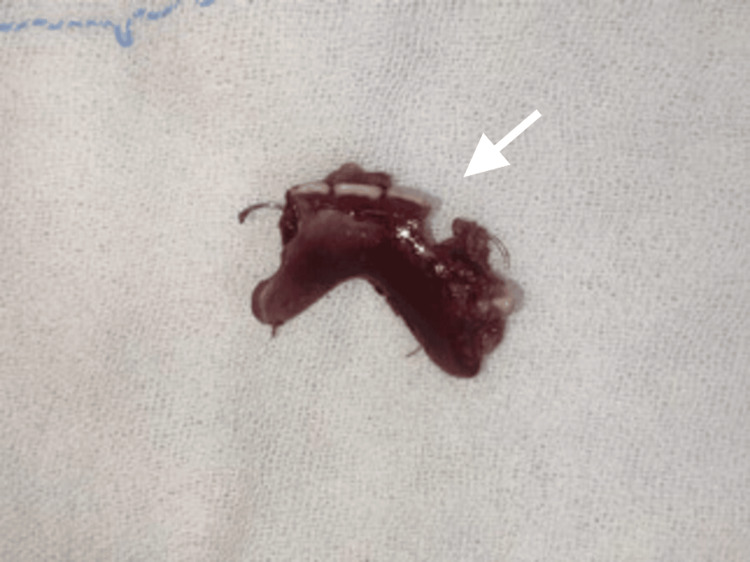
Close-up view of the ingested denture following retrieval.

After careful dissection, an intact partial denture, along with the ingested cotton, was identified within the jejunal lumen (Figure 3) and was successfully extracted (Figure 4).

Due to the presence of multiple perforations in a short, unhealthy jejunal segment, trimming and primary closure were deemed risky. Therefore, a decision was made intraoperatively to perform a resection of 10 cm of the jejunum, along with removal of the foreign bodies, followed by an end-to-end jejunojejunal anastomosis using hand-sewn full-thickness simple interrupted sutures. Thorough peritoneal lavage was performed, and a surgical drain was placed before closure.

Postoperatively, the patient received parenteral antibiotics and experienced an uneventful recovery. Oral feeding was initiated on postoperative day five, and the patient was discharged in stable condition on day seven. Unfortunately, the patient was lost to follow-up after discharge; therefore, medium- and long-term follow-up data were not available.

## Discussion

Foreign body ingestion is a relatively common occurrence in children aged six months to six years. However, adults can also accidentally swallow foreign bodies, especially if they have certain gastrointestinal tract conditions or risk factors such as advanced age (as demonstrated in this case), loss of sensation with dental prostheses, hurried eating habits, or improper food preparation [7]. Epidemiological data regarding the prevalence and incidence of foreign body ingestion in adults remains limited. Available studies indicate that the most commonly swallowed foreign bodies by adults are fish bones (9%-45%), bones (8%-40%), and dentures (4%-18%) [1]. The most common foreign body that causes bowel perforation varies from one study to another. Madrona et al. reported chicken bones as the most common cause [8], while Goh et al. concluded that fish bones are the leading etiology [9].

Dental prostheses, such as dentures, are typically made of a radiolucent composite resin called polymethylmethacrylate, with porcelain teeth. The metallic frameworks, clasps, or wire retainers of dentures are the only radiopaque components. Dentures with metallic frameworks have a higher reported incidence of impaction and perforation. Due to the predominantly radiolucent nature of dentures, they can be easily overlooked in plain radiographs, necessitating a high level of suspicion for diagnosis. While a plain X-ray is recommended as the initial investigation, multiplanar CT scans are the preferred choice to precisely locate the ingested denture [10].

A normal white blood cell count does not exclude jejunal perforation, particularly in elderly patients, in whom the inflammatory response may be attenuated [11]. Moreover, early or localized perforation may fail to elicit an immediate leukocytic response. This case underscores an important limitation of relying solely on laboratory markers to exclude serious intra-abdominal pathology and highlights the pivotal role of clinical judgment and cross-sectional imaging in establishing the diagnosis.

The esophagus is the most common site for foreign body impaction. Once in the stomach, dentures will spontaneously pass out in 80%-90% of cases [12]. Although rare, denture impaction can also occur in the large bowel. Most swallowed dentures that pass through the ileocecal valve should theoretically traverse the large bowel without difficulty. The solid consistency of fecal content may even provide some protection against trauma to the large bowel [13]. However, cases of denture impaction in the small bowel are extremely rare and limited in number.

In most cases, a swallowed foreign body will pass through the digestive system spontaneously within three to five days without complications. However, if the foreign body does not pass, complications, such as perforation, may occur within a week [14]. A recent review concluded that both hooked and unhooked dentures carry an equal risk of complications, including perforation, once they become impacted [15]. Perforation associated with pointed foreign bodies typically occurs in angulated segments of the bowel, such as the rectosigmoid or ileocecal junction. However, it may occur at any site along the gastrointestinal tract [5]. The most common site of perforation reported is the terminal ileum (38.6%) due to its narrow lumen, according to Goh et al [9]. Rare perforation sites include the appendix, hernia sac, and Meckel diverticulum. In the case presented, the perforation was found one meter distal to the duodenojejunal flexure.

Bowel obstruction or perforation caused by swallowed foreign bodies can present with various symptoms and signs. In this case, acute abdominal pain and vomiting were the main presenting symptoms. Other documented signs and symptoms include inflammatory masses, omental pseudotumor, localized abdominal abscess, intra-abdominal bleeding, and colovesical or enterovesical fistula.

Statistically, approximately 80% of swallowed foreign bodies will pass through the gastrointestinal tract spontaneously, while 10%-20% may require non-operative interventions, and 1% or less may require surgical management [1]. The current recommendation for managing swallowed sharp foreign bodies is initially conservative. Close observation and monitoring with serial imaging for a period of three days is suggested. In cases where conservative management fails, if the foreign body is located in the upper gastrointestinal tract, 70%-75% can be removed using rigid endoscopy, while a few cases may require surgical intervention [16]. However, in the case of lower gastrointestinal tract foreign bodies that fail to progress, early surgical intervention is recommended to prevent potential perforation and subsequent complications [17].

## Conclusions

Elderly patients with an acute abdomen and a history of denture ingestion should be suspected of experiencing complications related to foreign body impaction. It is important to conduct a contrast-enhanced CT scan to locate the foreign body and detect potential complications. In cases where the foreign body becomes impacted in the lower gastrointestinal tract and does not progress, early surgical intervention should be considered for its removal. Delaying surgical management in such cases can significantly worsen the condition and expose the patient to potentially life-threatening complications, including bowel perforation.
